# Physical Fitness and Locomotor Skills in Children With Esophageal Atresia-A Case Control Pilot Study

**DOI:** 10.3389/fped.2018.00337

**Published:** 2018-11-06

**Authors:** Tatjana T. König, Oliver J. Muensterer

**Affiliations:** Department of Pediatric Surgery, Universitätsmedizin Mainz, Mainz, Germany

**Keywords:** fitness, physical activity, congenital malformation, esophageal atresia, motor function, gastroesophageal reflux

## Abstract

**Background:** Patients with esophageal atresia (EA) often experience physical limitations. With increasing survival over the past decades, the focus in care shifted toward improving the long-term quality of life. We performed validated testing fitness and motor skills in children born with EA.

**Methods:** Patients with EA were evaluated using the standardized *Kinderturntest Plus/ Deutscher Motorik Test* after caregiver's written consent. Test scores range from 1 to 5 (compared to an age- and gender matched standard population). Caregivers completed an online-questionnaire on patient history.

**Results:** Seventeen patients (median age 7 [3–12] years) were included. Comorbidities were prematurity (54%), birth weight <1,500 g (23%), congenital heart disease (46%), developmental delay (38%), skeletal deformity (23%), and anorectal malformation (15%). The mean test score was significantly lower in children with EA (2.19) compared to a control group matched for age, gender, body weight, and –height, (2.75, *p* = 0.04), and the general population (3, *p* = 0.00). Distribution of patient scores was below the 41st performance percentile for gender and age in 54–63%. Caregivers identified notable deficits of strength and endurance. All but one patient complained about discomfort during physical exercise, most commonly respiratory distress (46%) and gastroesophageal reflux symptoms (31%). Notably, 93% of subjects participated in regular school physical education classes, and 86% participated regularly in additional organized sporting activities.

**Conclusions:** Children after EA repair have decreased physical fitness and impaired locomotor function compared to the general population on a standardized test. Physical discomfort is frequent during exertion. To avoid demotivation, locomotor skill should be promoted at each individual's comfortable level.

The study was registered at www.researchregistry.com (No. 3707).

## Background

Even after successful primary repair, patients born with EA often experience and learn to live with a broad spectrum of physical limitations (1). With increasing survival over the past few decades, the focus in care is shifting toward improving the long term quality of life. For most children, physical activity and sports participation are important pillars of physical health, social engagement, and emotional well-being (2). Previous studies have shown significantly reduced spirometric exercise capacity and total lung capacity in children after EA repair (3, 4). Furthermore, spirometry revealed airway obstruction in about half of EA patients, correlating well with clinical symptoms (5). Harmsen et al showed a reduced score of children with EA in the M-ABC, a motor test of handedness, ball- and balance skills, at the ages of 5 and 8 years (6). In general, impaired motor function has also been found in 5-year-olds with major anatomical congenital anomalies (7) or other chronic health conditions (8). Since many children with EA have associated anomalies, it remains unclear whether these physical impairments are primarily due to the EA itself or to comorbidities such as congenital heart disease (CHD). Also, it is unknown if caregivers consciously or subconsciously hold back patients with EA due to increased perceived risks and if decreased fitness and motor skills are a secondary phenomenon.

In order to investigate the role of esophageal atresia, symptoms during physical activity, motor skills, and sports participation, we evaluated a cohort of esophageal atresia patients during the annual meeting of the national patient support group using a validated motor skills test and correlated these findings to the information given by the caregivers in a brief questionnaire.

## Materials and methods

### Setting

A playful, voluntary extracurricular activity for children was established at the annual meeting of the German national patient support group for patients with esophageal atresia *KEKS e. V*. At our hospital playground, an activity parcours was built, that included items of the *Kinderturntest Plus/Deutscher Motorik Test* (KTT/ DMT, see below), a standardized motor test. The event started with a warm-up program, and children had the opportunity to run and play in between the tests. Every participant received a certificate and a gift bag after completing the tests.

### Standardized testing

**Figure 1:**
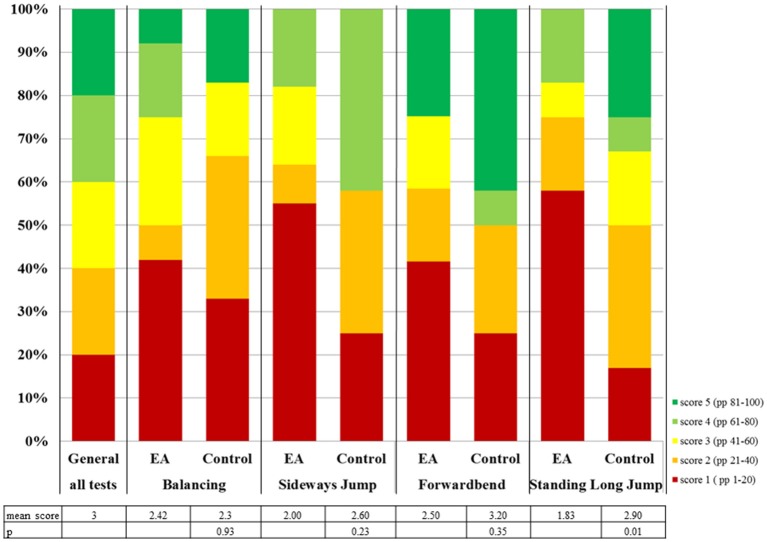
Distribution of locomotor test scores of children with EA compared to the general population and control group. The distribution of test results for the general population is the same for every test item. PP = performance percentile.

The *Kinderturntest Plus (KTT)* (9) is an extension of the *Deutscher Motorik Test (DMT)* (10) for younger children. While the *DMT* provides standardized data for boys and girls ages 6–18, the *KTT* is also applicable for children 4 and 5 years of age. The scores for each test item range from 1 to 5 based on the performance percentile of the test result of a healthy German standard population matched for gender and age (Figure 1, “General”). Four test items are contained in both the *KTT* and *DMT*, and therefore applicable for all children 4 years or older.

#### Balance

Participants balance backwards on three bars with decreasing width (6, 4.5, and 3 cm wide, each 3 m long), counting the steps completed backwards without touching the ground. There are two attempts on each bar.

#### Coordination

To test motor coordination under time constraint, participants jump sideways back and forth from two adjacent squares (50 × 50 cm each) for 15 s as many times as possible. Jumps completed without touching the lines are counted and scored.

#### Strength

Strength is tested by standing long jump. Participants stand behind a line and, without taking an extra step, jump as far as they can. Distance between the starting line and the back heel is measured perpendicular to the line.

#### Flexibility

Participants stand on a step, toes close to the edge with straight knees, and bend forwards. The distance between the fingertips and the edge is measured.

### Questionnaire

Parents received a link to an online-questionnaire on their child's medical history, physical activity, and symptoms during workout to answer at home (see Appendix for complete questionnaire). The test results and data from the questionnaire were used to give an individual activity recommendation for each participant. Questions 5, 14, 15, and 16 (marked with a ^*^) were adapted from the standardized German Health Survey for Children and Adolescents (*KiGGS*) (11) questionnaire to facilitate comparison to the national cohort of healthy individuals.

### Statistics

EA participants received their test scores for each item. The test scores of the *KTT/DMT* are based on gender- and age-based performance quintiles and are therefore not normally distributed (Figure 1, 1st column). A separate cohort of German school children from the nationwide *Motorik-Modul Baseline* study, conducted by the Karlsruhe Institute of Technology (12), was used as a representative sample of the general German population (*n* = 4529, 4–17 years). The dataset comprises biometric data and motor test results. Medical records were not collected in the study, since healthy children were tested in a school environment. The performance percentiles for each motor task and, consequently, the scoring system of the *KTT/DMT* is based on this source data. Therefore, in this cohort, the mean score for each item and the overall mean score of test results is 3 by definition. Out of this sample, each EA participant from our own cohort was matched with a healthy control from the *Motorik-Modul Baseline* study (Table 1). The matching was performed for age, gender, height and weight in this order. In order to rule out the impact of low bodyweight and –height, the EA motor test scores were compared to both the motor test scores of the matched controls and the mean result of the general population using the Wilcoxin-Sign-Rank-Test. In order to investigate the differences between EA patients with further comorbidities and symptoms, the Kruscal-Wallis-Test was used.

**Table 1:** Participants and Test scores according to *KTT/ DMT* and matched controls (1 = PP 1–20, 2 = PP 21–40, 3 = PP 41–60, 4 = PP 61–80, 5 = PP 81–100), PP = performance percentile, SWJ = sideways jump, SLJ = standing long jump, BMI = Body-Mass-Index, BMI-SDS = body-mass-index standard-deviation-score, m = male, f = female, ^*^outlier.

**Age, gender**	**Height [cm]**	**Height z-score**	**Weight [kg]**	**Weight z-score**	**BMI**	**BMI-SDS**	**Balance score**	**SWJ score**	**Flexibility score**	**SLJ score**
**ESOPHAGEAL ATRESIA**										
4, m^*^	92	−3,64	10	−5,43	11,81	−3,8	3	3	2	1
4, m	104	−1,06	15	−1,59	13,87	−1,25	2	4	1	4
4, m	110	0,01	18,4	0,01	15,21	−0,17	3	0	5	3
5, m	105	−2,22	16	−2,04	14,51	−0,89	3	1	3	1
6, m	112	−2,01	16,7	−2,67	13,31	−2,24	4	1	1	1
7, f	120	−1,25	18,4	−2,26	12,78	−2,14	1	1	1	1
8, m	128	−1,04	24,5	−1,2	14,95	−0,74	1	1	1	1
9, f	128	−1,75	21,6	−2,58	13,18	−2,13	1	2	5	2
9, m	130	−1,54	22,7	−2,52	13,43	−2,14	1	1	1	1
10, f	146	0,41	27	−1,64	12,67	−3,12	5	1	5	4
11, f	143	−0,82	35	−0,82	17,12	−0,35	4	4	3	2
12, f	151	−0,89	56,5	0,98	24,78	1,65	1	3	2	1
**CONTROL GROUP**										
**Age, gender**	**Height [cm]**	**Height z-score**	**Weight [kg]**	**Weight z-score**	**BMI**	**BMI-SDS**	**Balance score**	**SWJ score**	**Flexibility score**	**SLJ score**
4, m	95,8	−2,83	14,6	−1,81	15,9	0,34	3	4	5	2
4, m	104,5	−0,95	15,1	−1,53	13,8	−1,64	5	4	5	4
4, m	110,4	0,32	18,5	0,05	15,2	−0,15	3	4	5	5
5, m	106,2	−1,98	16,8	−1,62	14,9	−0,36	2	4	5	3
6, m	113,1	−1,81	17,6	−2,18	13,8	−1,63	2	2	2	2
7, f	120,1	−1,23	20,1	−1,58	13,9	−1,26	1	2	2	2
8, m	128,9	−0,89	23,3	−1,58	14,0	−1,45	2	4	4	5
9, f	128,8	−1,63	22,9	−2,17	13,8	−1,67	1	1	1	1
9, m	129,4	−1,63	22,6	−2,55	13,5	−2,06	5	2	2	2
10, f	147,9	0,41	32,1	−0,64	14,7	−1,3	2	1	1	5
11, f	143,4	−1,09	35,5	−0,74	17,3	−0,26	1	2	5	3
12, f	152,5	−0,68	57,8	1,09	24,9	1,67	1	1	1	1

## Results

### Motor test

**Figure 2:**
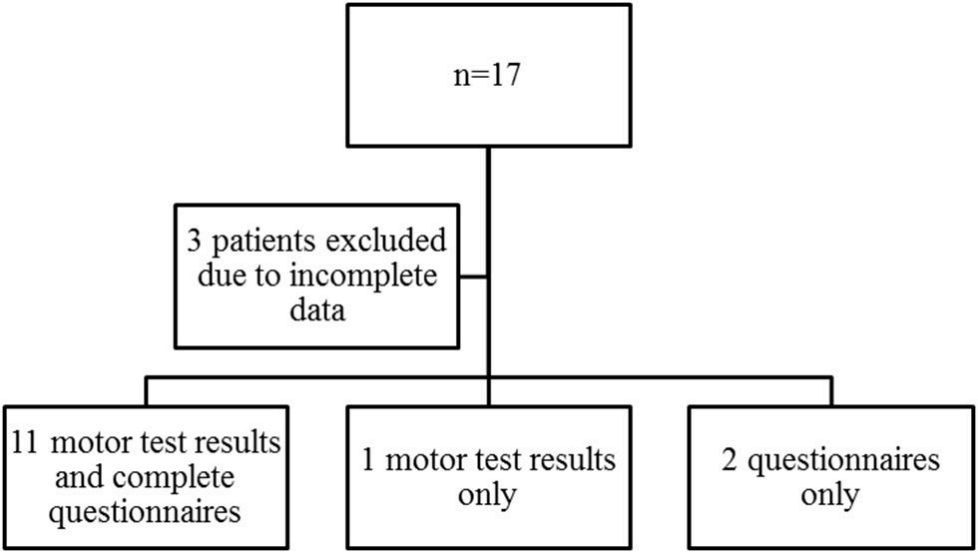
Inclusion Flowchart.

A total of 17 children with EA were evaluated for locomotor skills. The median age was 7 (range 3–12) years (9 boys, 8 girls). Three patients hat to be excluded due to incomplete data, the motor test results of two 3-year-old children could not be evaluated because of missing reference values in that age group, but the data of the questionnaires was included. For one participant, parents didn't complete the questionnaire (Figure 2).

Mean locomotor skill test scores in the EA cohort were below the average of three for all tested items (Figure 1). The mean test score for the EA cohort (2.19) was significantly lower than the control group (2.75, *p* = 0.04) or the general population (3, *p* = 0.00). There is a trend to a lower mean test score for EA patients compared to the controls for all tested items (Figure 1). For standing long jump, the mean score was significantly lower in children with EA (1.83) compared to the control group (2.9, *p* = 0.01) and the general population (3.00, *p* = 0.01). For sideways jumps only the difference between EA (2.00) and the general population was significant (3, *p* = 0.01). EA participants' results were below the 41st performance percentile (scores 1 or 2) in 54–63% (Figure 1), much more frequent than expected.

The children showed low bodyweight and -height according to German growth charts. The mean z-score was −1.81 (SD 1.59) for weight, −1.32 (SD 1.06) for height and −1.44 (SD 1.47) for body-mass-index (BMI, Table 1). The mean body weight (z-score −1.3, SD 1.0) and –height (−1.2, SD 0.9) of the matched controls was higher than in the EA group. The patients were matched for height first and then the control with the best match for weight was chosen, which tended to be higher rather than lower in the EA group. However, one extreme outlier had the biggest impact on the differences (Table 1, ^*^).

There was no difference in the test scores of younger children (<8 years, *n* = 6, mean score 2.23) or the older group (≥8 years, *n* = 6, mean score 2.21).

### Questionnaire

According to the parents, 86% of subjects enjoyed engaging in physical activity, and 93% participated in regular physical education programs in kindergarten or school. However, caregivers subjectively reported a deficit in locomotor skills compared to class mates: children are “most of the time” or “always” able to keep up with class mates in 12% regarding speed, and 8% regarding both strength and endurance. Eighty-six percent participated in sports outside school in a sports club at least once a week, but only 29% were also physically active in their free time without organized sports programs.

**Figure 3:**
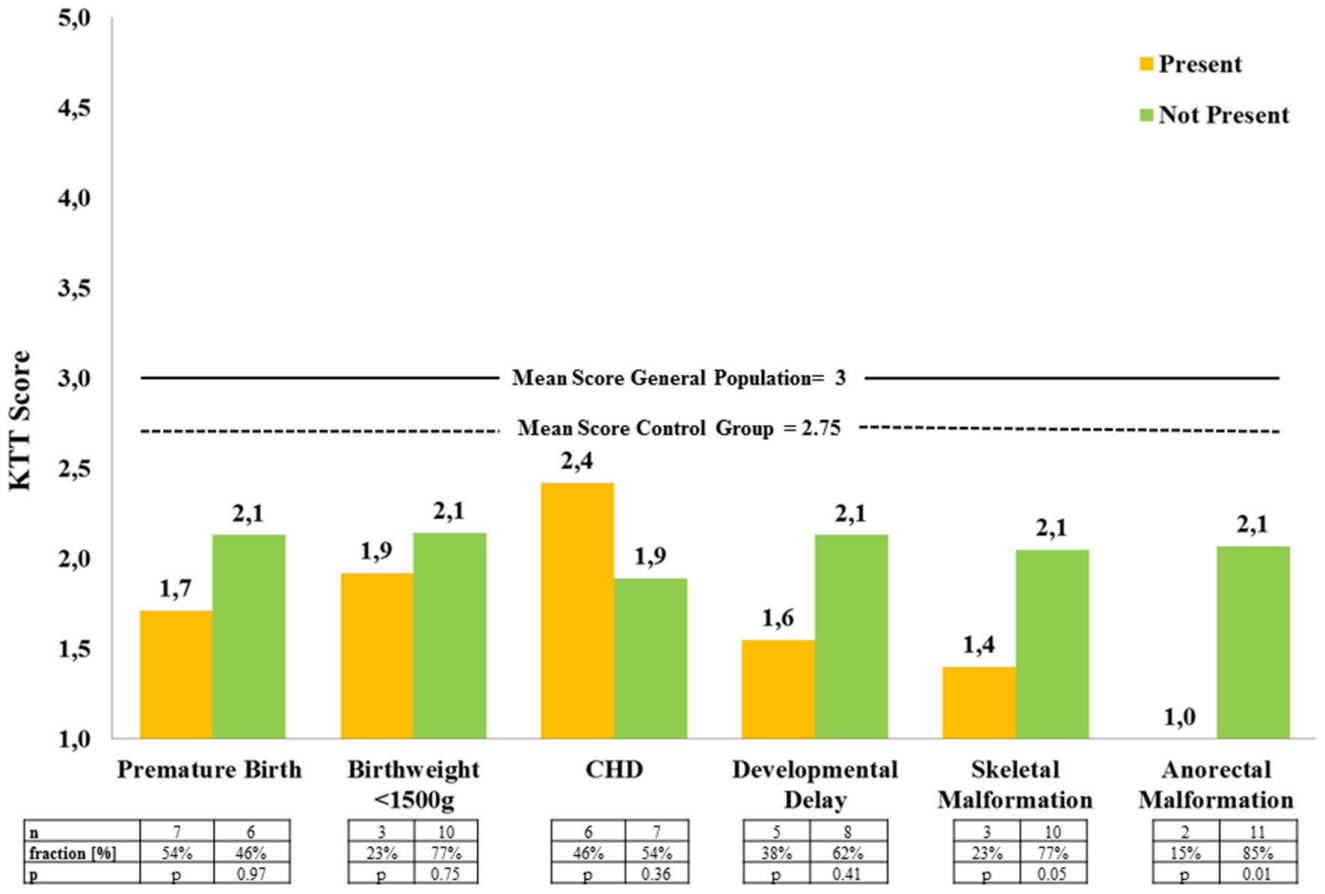
Mean locomotor test results of EA patients with and without associated comorbidity (*KTT* = *Kinderturntest Plus*, CHD = congenital heart disease).

Comorbidities identified in the study cohort were prematurity (54%), birth weight <1,500 g (23%), CHD (46%), developmental delay (38%), skeletal deformity (28%), and anorectal malformation (15%, Figure 3). Children with additional comorbidity, except for CHD, had lower mean motor test results (Figure 3). This trend was only significant for anorectal malformation (Figure 3).

**Figure 4:**
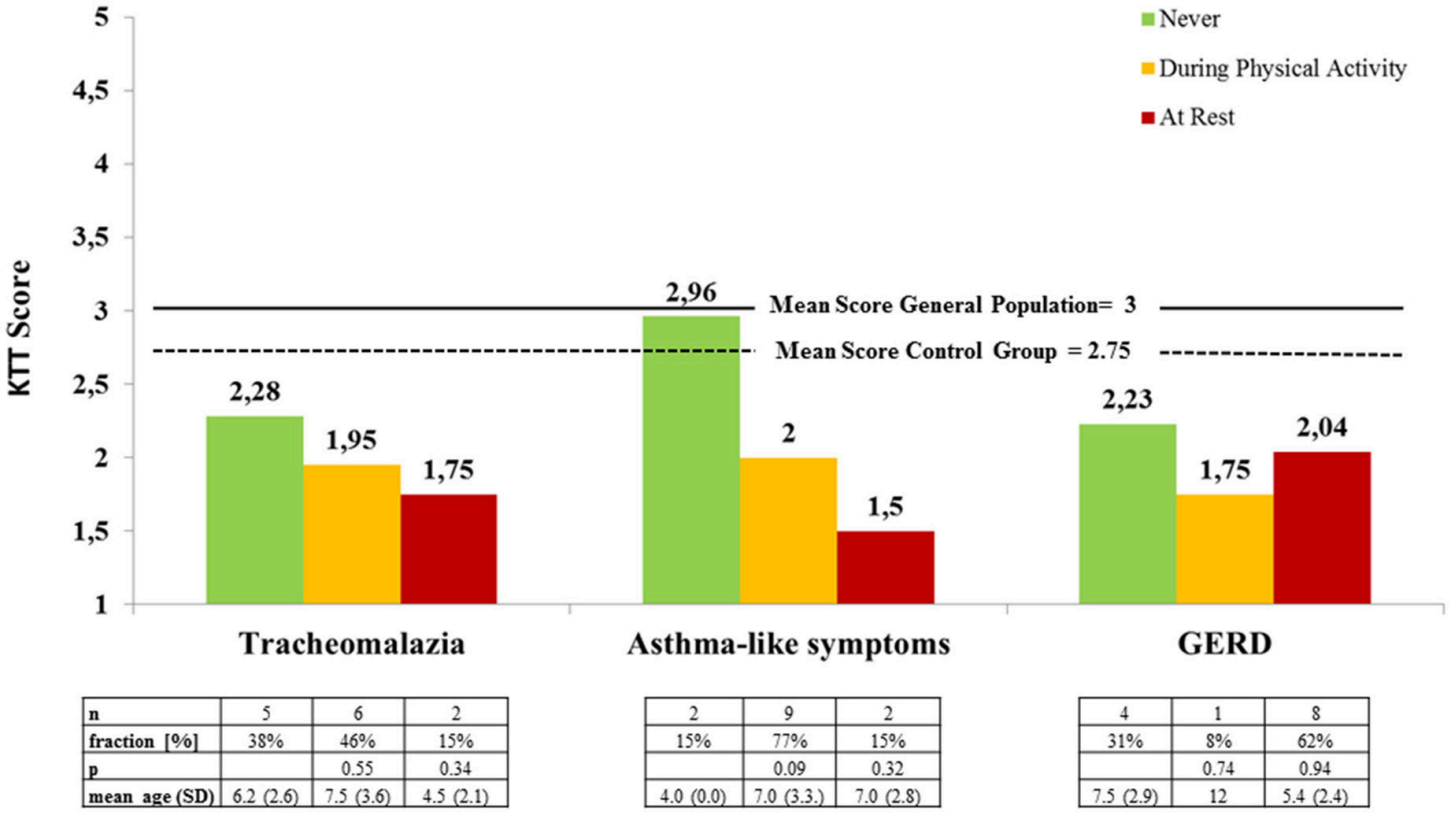
Mean locomotor test results of EA patients according to perceived symptoms during physical activity and at rest (*KTT* = *Kinderturntest Plus*).

Subjective symptoms of respiratory distress (RD) and gastroesophageal reflux disease (GERD) were the most common complaints and caused the greatest limitation to physical activity and athletic performance according to the parents (RD 46%, GERD 31%). Even though the differences are not significant, there is a clear trend toward better overall motor test scores at the absence of tracheomalazia and broncho-pulmonary obstruction (“asthma-like symptoms,” Figure 4). According to caregivers, respiratory symptoms increased with the level of exertion (Figure 4 below). Symptoms of GERD were reported in 62% of patients at rest and an additional 8% only during physical activity (Figure 4). EA patients with or without subjective symptoms of GERD showed hardly any difference in the motor test results (Figure 4). Most frequently, subjective symptoms occurred during sprinting (38%) and great exertion of strength (31%).

None of our patients complained of musculoskeletal pain during exercise. Only one patient never complained about any exercise-associated discomfort at all. None of the caregivers reported complaints during jumping on the trampoline (Table 2).

**Table 2:** Frequency of symptoms during various activities according to parent.

**Activity**	**Never/sometimes**	**Often/always**
Jumping on trampoline	99%	0%
Run fast	62%	38%
Great exertion of strength	69%	31%
Upside-down position	76%	23%
Riding bicycles	84%	15%
Jogging	76%	23%

## Discussion

Physical activity, sports, and play are a major health promotor and essential for normal emotional, cognitive, and psychosocial development of children and adolescents (13, 14). According to the World Health Organization (WHO), growing physical inactivity has become the fourth leading risk factor for mortality worldwide (15). For an optimal health outcome, the WHO recommends at least 1 h of daily moderate to vigorous physical activity, 12,000 steps, and a restriction of seated time to 2 h daily for children (15). Only 11 to 35 percent of the healthy German population fulfills these recommendations (16). Given that physical activity and fundamental motor skills during early childhood determine physical activity in later life (17), there is concern that these findings will have adverse impact on the overall health in the future.

Promotion of physical activity is particularly important for children with pre-existing health conditions. Children with chronic disease generally show a decreased exercise capacity that might be due to physical inactivity caused by prolonged hospitalization, recurrent infection and growth retardation during early childhood (4, 7). Esophageal atresia is a complex health-condition that adversely affects the gastrointestinal and respiratory system. One would expect that children with esophageal atresia therefore are limited in their ability to enjoy organized sports. Surprisingly, more children (86%) in our study participated in an organized sports program at least once a week compared to published reference groups in the general German population (53%) (16). Also, the vast majority of children (93%) participated in regular school physical education classes. This finding is consistent with a survey by Dellenmark-Blom et al. in which only 8.5% of 1,371 patients with EA could not participate regularly in sports and play (1). While participation in organized sports was high in our study, only 28% of participants reported playing sports in their free time on their own or with friends, compared to a reported 50% benchmark in the general population (17).

Our data showed a mean motor test score substantially below average for all tested items. Significantly lower mean scores were recorded in the standing long jump, which is generally considered a measure of strength. In young children, motor coordination may also be tested in this complex task. Consistent with our measurements, parents described a lack of muscular strength in comparison to classmates. The mean score for sideways jumps, testing for coordination pressed for time, was significantly lower in EA patients compared to the general population. Interestingly, balance and flexibility were decreased as well, even though both of these skills do not depend on either exercise capacity or muscular strength and, at least theoretically, should therefore be less compromised in children with EA. A general lack of physical activity might contribute to deficits in these fields of motor skills. Our findings corroborate the study by Harmsen et al. which also describes compromised balance skills in children with EA at the ages of 5 and 8 years. In this group, improved skills could be shown at the age of 8 years, if the children engaged in physical activity during the observation period (6). This study shows that children with EA are trainable, and emphasizes the importance of promotion regular physical activity.

Growth retardation is a common finding in patients with EA and has also been seen in our patients. All children had a negative z-score for height and all but one a negative z-score for weight. According to parents, a low body weight and –height compared to class mates influence the motor performance, especially in terms of muscular strength. However, the control group got an average mean result in spite of their low body weight and –height. Generally, being smaller and having a lower body weight than peers makes it hard to compete in physical education or organized sports. Growth retardation has also been described as a cause for low self-esteem if children are teased by peers (1). To be successful in athletic competition, children may be more comfortable in sports in which athletes compete in weight categories or light body weight is an advantage.

In our data, there was a trend toward better motor test results in absence of comorbidity, except for patients with CHD without reaching statistical significance. CHD, however, was not further classified. There were two children with EA and anal atresia. Both of them had the lowest possible motor mean score (1.00), which was significantly lower than the mean score of the rest of the EA group in spite of the small number (Figure 3). Looking at these two patients more closely, VACTERL association was present in one case, and the additional CHD and coloboma in the other. Postoperative scoliosis, scapula deformity, and chest wall deformity are frequent in children with EA after neonatal thoracotomy. Despite the high incidence, postoperative skeletal deformities rarely lead to functional impairment or even require treatment (18). In our patients, no postoperative scoliosis or chest deformity with subjective impact on physical activity was found. Congenital skeletal deformity was present in 28%. These children had a lower mean motor test score than the other EA participants. In general, more specified data and a bigger cohort are needed to support the impact of comorbidity on motor function in EA patients.

In our study, most common complaints during exercise were respiratory symptoms and gastroesophageal reflux disease, which occurred mainly during vigorous activity like running or great exertion of strength. Chronic lung disease, GERD and growth retardation have also been described as main chronic morbidities in children with EA by other authors (1, 4, 19). Each of these conditions has a negative impact on athletic performance. Coaches, teachers, pediatricians, and caregivers should be aware of these findings and provide appropriate support.

Respiratory issues, such as tracheomalazia, irreversible non-allergic airway obstruction and pulmonary restriction, caused by recurrent infection and microaspiration are common in patients with EA (3, 4). Acute respiratory problems most frequently occur in early childhood, leading to chronic bronchitis, obstructive and restrictive ventilatory defects in later life (3). In our patients, no relation between the severity of respiratory symptoms and age could be shown (Figure 4). During or even after exercise, increased ventilation leads to airway dehydration, that may induce bronchial constriction in predisposed individuals. With improving exercise capacity, respiratory rate decreases and symptoms may cease (20). An adequate preexercise warm-up is substantial in patients with bronchial obstruction. Other measures to minimize respiratory stress may include adequate use of inhalatory therapy, avoiding sudden changes of temperature, cold (<20°C), very dry or polluted air, and high allergen concentration (20). Endurance *per se*, which is closely related to lung function, was not tested in our trial, but parents reported a substantial lack of endurance in comparison to peers in the survey. In a treadmill exercise trial by Dittrich et al. patients with EA exhausted early, those with restrictive ventilatory defects sooner than those with obstructive ventilatory defects. Though clinical symptoms seem to improve with age, lung function tends to get worse (3). Exercise capacity has also been shown to be significantly decreased in patients with EA. However, according to one study, structural lung problems *per se* did not account for a decreased exercise capacity in these patients (4). Exercise programs may include primarily aerobic training, and respiratory muscle training (18).

Symptoms of GERD occur to some extend in almost all patients with EA (1, 19). GERD is also considered as one of the pathologies leading to chronic airway inflammation, even further compromising athletic performance. In our study, severe GERD was present in 62% of patients at rest. During physical activity, severity of symptoms increased qualitatively, according to caregivers. In half of these patients, symptoms of GERD subjectively caused the greatest limitation to physical activity and athletic performance. Esophageal peristalsis, pressure of the lower esophageal sphincter, the angle of His and the pincer-like crural part of the diaphragm create natural barriers for gastroesophageal reflux. All these factors are compromised in patients with EA. In addition, gastric emptying is delayed due to antral hypomobility in patients with EA (21). In patients without GERD at rest, physical activity did not lead to symptoms in our cohort. Hence, while the subset of EA patients that did not have GERD at rest seem to be unsusceptible to exercise-induced GERD, reflux symptoms seemed to be exacerbated by exercise in the others.

Symptoms of GERD account for most upper gastrointestinal symptoms in healthy athletes and worsen with the intensity of physical activity and during postprandial activity. Especially during high intensity workout [maximal oxygen uptake (VO_2_ max) >90%], the number and duration of reflux episodes increase by decreased esophageal motility, decreased pressure of the lower esophageal sphincter (22, 23), increased intraabdominal pressure, decreased intestinal blood flow, and body positioning (22, 23). Aerobic training improves all of these these mechanisms. Anti-reflux surgery in EA patients had no effect on lung function in childhood (4), its effect on symptoms of GERD during exercise remains unclear. Further studies should address whether anti-reflux measures lead to increased participation and enjoyment of sports activity in those patients affected by reflux symptoms. In the meantime, physicians and pediatric surgeons should consider prescribing antacids for those experiencing reflux symptoms during physical exertion. Some practitioners recommend avoiding a solid, high fat or fiber diet prior to vigorous physical activity (22).

In contrast to our expectations, none of the parents reported any symptoms during jumping on the trampoline (Table 2). A possible explanation is that perception of symptoms might be altered by the factor “fun” during physical activity, which in itself is an important tool in promoting exercise in these children.

Our study has some limitations. For one, there were only 12 patients who qualified for final analysis of motor results because data on the others were incomplete. The data for the control group was not collected in our own hospital, but was obtained from the source data of the *Motorik-Modul Baseline* study (12) in collaboration with the Karlsruhe Institute of Technology. The dataset comprises biometric data and motor test results, but no medical records of participants, since healthy individuals were tested in a school setting. In our setting, participation was based on playful invitation, the children were left to decide themselves whether they would participate, and some simply didn't want to complete all items. There was no pressure, persuasion or coercion to participate or complete the parcours. While this approach limited the number of patients enrolled, we believe it decreased the bias of performing in a study environment (avoiding the Hawthorne effect). Patients had much comorbidity such as CHD and prematurity, that may contribute to motor performance but were not classified in detail. Also, other important factors, like open or thoracoscopic repair and anti-reflux surgery, should be addressed in future studies. The families attending the KEKS e.V. annual meeting may represent a selected group of patients with EA because they may represent more severely affected cases. Conversely, families who participate in a parent-patient support group may also be more likely in actively engaging in their children's overall well-being.

## Conclusion

Children after EA repair have decreased physical fitness and impaired locomotor function compared to a control group and the general population on a standardized test. Physical discomfort is frequent during great exertion and athletic performance is mainly limited by symptoms of RD and GERD. These symptoms cause major subjective discomfort to the children and may lead to avoidance of physical activity in general. Aerobic training improves symptoms RD and GERD in other patients and might be helpful in patients with EA. Therefore, physicians should encourage caregivers to have their children engage in physical activity after optimizing training methods, dietary patterns, and environmental factors. Training balance, flexibility, motor coordination is mostly independent of symptoms of RD and GERD and should be encouraged as early as possible, since motor skills at a young age determine physical activity in later life. Endurance and muscular strength should be continuously promoted at a comfortable, but still challenging, level of exertion to improve fun and commitment and ensure an active and healthy life style for children and adults with EA. The effectiveness of such interventions should be assessed in future prospective studies.

## Data availability statement

The raw data of this study are available upon request from the lead author (TK). Reference values and test protocols for *Kinderturntest Plus* are available at www.badischer-turner-bund.de/jugend/kinderturnen/kinderturn-test-plus (last access 24.04.2018).

## Ethics approval and consent to participate

The ethics board of the state of Rhineland-Palatinate was consulted and approved of the study, exempting it from formal review due to exclusively anonymized data and minimal risk to the participants. All caregivers received information on the study and signed an informed consent.

## Author contributions

The study was planned, conducted, and managed by the first author (TK) with support of the second author (OM). Both authors were directly involved in the drafting, writing, and editing of this manuscript.

### Conflict of interest statement

The authors declare that the research was conducted in the absence of any commercial or financial relationships that could be construed as a potential conflict of interest.

## Abbreviations

BMI: body mass index
BMI-SDS: body mass index- standard deviation score
CHD: congenital heart disease
*DMT*: Deutscher Motorik Test
EA: esophageal atresia
FEV 1: forced exercise capacity in one second
GERD: gastroesophageal reflux disease
*KEKS e*.V: “Patienten- und Selbsthilfeorganisation für Kinder & Erwachsene mit kranker Speiseröhre“ national German support group for patients with esophageal atresia
KiGGS: *Kinder- und Jugendgesundheitssurvey* German Health Survey for Children and Adolescents
KTT: Kinderturntest Plus
PP: performance percentile
RD: respiratory distress
SD: standard deviation
VO_2_ max: maximal oxygen uptake
WHO: World Health Organization.
